
JOURNAL INFORMATION
==============================
NLM Title Abbreviation: Wirtschaftsdienst
Journal ID: Wirtschaftsdienst
NLM Journal ID: 101086612

Wirtschaftsdienst (Hamburg, Germany : 1949)

ISSN: 0043-6275

ARTICLE INFORMATION
==============================
PMCID: PMC7896176
PMID: 33642650
DOI: 10.1007/s10273-021-2857-x
Article ID: 2857
Article version: 1
Subjects: Ökonomische Trends

Einbruch der Transferströme: Einfluss der COVID-19-Pandemie auf den professionellen Fußball

Collapse of Transfer Flows: Impact of the COVID-19 Pandemic on Professional Football

Frick Bernd bernd.frick@upb.de
1
Mainus David mainus@hwwi.org
2
Schumacher Paul schumacher.paul@web.de
2
1 grid.5659.f 0000 0001 0940 2872 Fakultät für Wirtschaftswissenschaften, Universität Paderborn, Warburger Straße 100, 33098 Paderborn, Deutschland
2 grid.435054.3 0000 0001 2227 8589 Zweigniederlassung Bremen, HWWI, Fahrenheitstr. 1, 28359 Bremen, Deutschland
Electronic publication date: 2021 Feb 20
Print publication date: 2021
Volume: 101
Issue: 2
First page: 144
Last page: 146
Copyright: © Der/die Autor:in(nen) 2021
License: Open Access: Dieser Artikel wird unter der Creative Commons Namensnennung 4.0 International Lizenz veröffentlicht (http://creativecommons.org/licenses/by/4.0/deed.de).
Open Access wird durch die ZBW–Leibniz-Informationszentrum Wirtschaft gefördert.
License URL: https://creativecommons.org/licenses/by/4.0/

issue-copyright-statement: © The Author(s) 2021

==============================

Die pandemische Ausbreitung des SARS-CoV-2-Virus und die damit einhergehenden Kontakt- und Distanzierungsmaßnahmen hatten und haben schwerwiegende gesamtwirtschaftliche Folgen. Das weitgehende Verbot von kulturellen und sportlichen Großveranstaltungen hat zahlreiche Wirtschaftszweige vor neue Herausforderungen gestellt und beispielsweise den professionellen Sport schwer getroffen. Der professionelle Fußball, der von der Weltfinanzkrise 2008 bis 2009 weitestgehend verschont blieb, hat erstmals im 21. Jahrhundert drastische Einnahmerückgänge verbuchen müssen (Quitzau und Vöpel, 2020).

Die Saison 2019/2020 wurde in Europa erheblich durch Zwangsunterbrechungen beeinträchtigt. Ab Ende März 2020 beschlossen nahezu alle nationalen Verbände, dass eine Fortsetzung des Spielbetriebs nicht vertretbar sei; lediglich in Weißrussland wurde die Saison fortgesetzt (Bond et al., 2020). Nach einer mehrwöchigen Unterbrechung wurde der Spielbetrieb auch in vier der fünf Top-Ligen wieder aufgenommen.[1] Während der französische Verband die Saison vorzeitig für beendet erklärte, wurde der Wettbewerb in den Ligen in Deutschland, England, Italien und Spanien wieder aufgenommen. Die beiden internationalen europäischen Wettbewerbe, die Champions und die Europa League, wurden ebenfalls nach einer mehrmonatigen Pause in einem modifizierten Turniermodus zu Ende gespielt.

1 Als Top-5- oder Big-5-Ligen gelten die höchsten und zugleich einnahmestärksten Spielklassen der fünf besten europäischen Verbände in der Fünfjahreswertung der Union of European Football Associations ( UEFA, 2020).

## Ligabetrieb mit wenigen oder gar keinen Zuschauern

Die Wiederaufnahme des Ligabetriebes war an strikte Hygienebedingungen gebunden, die einen Teil- oder meist kompletten Ausschluss der Zuschauer aus den Stadien vorsahen. Somit entfielen die Spieltagserlöse, darunter Ticket-, Marketing- und Gastronomie-Einnahmen, während die Instandhaltungs- und Bewirtschaftungskosten größtenteils weiterhin anfielen.[2] Um den damit einhergehenden finanziellen Verlusten entgegenzuwirken zog eine Vielzahl von Vereinen Gehaltskürzungen oder -aufschübe bzw. Kurzarbeit in Betracht. Weiterhin hatten zahleiche Klubs einen Rückgang der Sponsoreneinnahmen zu verkraften, da Werbeverträge neu verhandelt werden mussten oder nicht verlängert wurden (Frick et al., 2020a). Aufgrund der Zwangsunterbrechungen, des Zuschauerausschlusses und der infolgedessen geminderten Attraktivität der Liveübertragungen kam es in mehreren Ligen zu Auseinandersetzungen über die Übertragungsrechte und Medienverträge. In Frankreich verweigerte beispielsweise das Kommunikationsunternehmen Mediapro nach dem Saisonabbruch die ausstehenden Zahlungen für die Spielzeit 2019/2020 und verhandelte einen frühzeitigen Ausstieg aus dem Vertrag, der die partiellen Übertragungsrechte der ersten und zweiten französischen Spielklassen bis 2024 garantieren sollte (Panja, 2020; Reuters, 2020).

Obgleich bislang noch keine abschließenden Zahlen vorliegen, stellen die Einnahmeverluste sowohl für die Vereine als auch die Verbände eine enorme Belastung dar, die noch dadurch verschärft wird, dass bislang keine Aussagen über die Rückkehr der Zuschauer gemacht werden können. Lediglich für die höchste französische Spielklasse, die Ligue 1, liegt eine erste Auswertung vor, welche die finanziellen Ausfälle durch den Saisonabbruch auf insgesamt über 600 Mio. Euro schätzt (Ernst & Young und Première Ligue, 2020).

2 Die Ligaspiele der meisten Klubs mussten weiterhin in den Stadien der ersten Mannschaften stattfinden, da Trainingsanlagen oder kleinere Stadien meist nicht die ligaspezifischen Anforderungen erfüllen, beispielsweise Torlinientechnik, Rasenheizung (für die deutsche Bundesliga: DFL, 2009).

Zum aktuellen Zeitpunkt liegen dementsprechend nur Prognosen zum Effekt der COVID-19-Pandemie auf die Fußballwirtschaft vor. Nichtsdestotrotz liefern detaillierte Analysen einiger branchenspezifischer Indikatoren bereits erste Erkenntnisse über die vermeintlichen wirtschaftlichen Folgen. Dies gilt beispielsweise für die Kerndaten des Transfermarkts, auf dem Spielerwechsel besiegelt werden, sei es durch die Auflösung eines laufenden Vertrags, eine Ausleihe oder durch die Zahlung einer Ablösesumme. Diese Transferzahlungen orientieren sich meist am Marktwert eines Spielers, der primär durch individuelle Leistungsdaten, die restliche Vertragslaufzeit und das individuelle Vermarktungspotenzial des jeweiligen Spielers bestimmt wird (Gerhards et al., 2014; Emrich et al. 2019). Das Gleichgewicht auf dem Transfermarkt, welches sich zeitweilig einstellt, ergibt sich demnach aus der Nachfrage sowie dem Angebot der jeweiligen Vereine. Durch die steigende Unsicherheit und abnehmende Investitionsbereitschaft im Zuge der COVID-19-Pandemie scheint ein geringeres Transfervolumen somit als sehr wahrscheinlich.

## Transferausgaben sinken

Frick et al. (2020b) prüfen diese Hypothese mit Hilfe einer Regressionsanalyse mit den Transferzahlungen der Clubs der fünf bestplatzierten europäischen Ligen in den Sommertransferfenstern 2017, 2018, 2019 und 2020 als der abhängigen Variable. Die Ergebnisse dokumentieren einen massiven Rückgang der aggregierten Transferausgaben sowie -einnahmen im Sommer 2020 und sind in Tabelle 1 dargestellt.

Im Vergleich zum Transfersommer 2017 gingen die Ausgaben für Spielerneuverpflichtungen im Sommer 2020 je Club um 13 Mio. Euro zurück. Gemessen am Transferfenster 2019 ist der Einbruch der Ablösezahlungen mit einem Rückgang um 22 Mio. Euro noch ausgeprägter. Die Transfereinnahmen gingen im Vergleich zur Referenzperiode ebenfalls signifikant zurück, doch war die absolute Differenz hier mit 13 Mio. Euro etwas geringer. Im Vergleich mit dem Wechselzeitraum im Sommer 2019 fielen die Einnahmen im Sommer 2020 um 19 Mio. Euro geringer aus.

Weiterhin werden insbesondere für gezahlte Ablösesummen deutliche saisonübergreifende Unterschiede zwischen den Ligen deutlich. Die Werte der spanischen, französischen, deutschen und italienischen Clubs weichen signifikant von den höheren Ausgaben der Vereine der Premier League ab. Zudem wird ersichtlich, dass die sportlichen Leistungen das Transferverhalten erheblich beeinflussen. Eine schlechtere Platzierung in der nationalen Liga hat einen signifikant negativen Einfluss sowohl auf die Transfereinnahmen wie auch die -ausgaben, die mit jedem Tabellenplatz um 1,5 Mio. Euro geringer ausfallen. Zugleich weisen Vereine, die in einem der europäischen Pokalwettbewerbe vertreten sind, signifikant höhere Transfereinnahmen sowie -ausgaben auf. So investieren Champions-League-Teilnehmer in jeder Saison rund 62 Mio. Euro mehr in Neuverpflichtungen als die nicht für diesen Wettbewerb qualifizierten Teams, nehmen aber nur 39 Mio. Euro mehr für Spielerverkäufe ein als die nicht-qualifizierten Konkurrenten aus der jeweiligen nati-

Tabelle 1

**Regressionsergebnisse: Ablösesummen der Vereine der fünf höchsten europäischen Ligen**

Sommertransferfenster 2017 bis 2020

| | (1.1) OLS | (1.2) RE | (2.1) OLS | (2.2) RE |
|---|---|---|---|---|
| | Ablösewert neue Spieler (gekauft und geliehen) | | Ablösewert abgegebene Spieler (verkauft und verliehen) | |
| 2017 | Referenzsaison | | | |
| 2018 | -0,369<br>(4,657) | -0,369<br>(4,657) | -0,891<br>(4,274) | -0,891<br>(4,274) |
| 2019 | 9,137<br>(5,890) | 9,137<br>(5,890) | 4,772<br>(4,773) | 4,772<br>(4,773) |
| 2020 | -13,28***<br>(4,306) | -13,28***<br>(4,306) | -13,05***<br>(4,186) | -13,05***<br>(4,186) |
| Premier League | Referenzliga | | | |
| La Liga | -33,93***<br>(8,331) | -33,93***<br>(8,331) | 4,795<br>(6,957) | 4,795<br>(6,957) |
| Serie A | -22,36***<br>(8,428) | -22,36***<br>(8,428) | 9,432<br>(5,926) | 9,432<br>(5,926) |
| Ligue 1 | -40,50***<br>(7,442) | -40,50***<br>(7,442) | 2,076<br>(8,086) | 2,076<br>(8,086) |
| Bundesliga | -46,64***<br>(6,996) | -46,64***<br>(6,996) | -8,012<br>(5,334) | -8,012<br>(5,334) |
| Abschlussplatzierung | -1,476***<br>(0,511) | -1,476***<br>(0,511) | -1,614***<br>(0,490) | -1,614***<br>(0,490) |
| Aufsteiger (1 = ja) | -1,252<br>(4,784) | -1,252<br>(4,784) | -11,86***<br>(3,328) | -11,57***<br>(3,328) |
| Champions League (1 = ja) | 62,14***<br>(9,581) | 62,14***<br>(9,581) | 39,05***<br>(10,85) | 39,05***<br>(10,85) |
| Europa League (1 = ja) | 17,81***<br>(5,807) | 17,81***<br>(5,807) | 12,06**<br>(5,599) | 12,06**<br>(5,599) |
| Konstante | 76,87***<br>(10,01) | 76,87***<br>(10,01) | 43,87***<br>(8,012) | 43,87***<br>(8,012) |
| N Beobachtungen | 392 | 392 | 392 | 392 |
| N Teams | --- | 131 | --- | 131 |
| Beobachtungen je Team | --- | 1 bis 4 | --- | 1 bis 4 |
| R2*100 (insgesamt) | 47,4 | 47,4 | 39,6 | 36,6 |
| R2*100 (innerhalb) | --- | 4,2 | --- | 6,6 |
| R2*100 (zwischen) | --- | 69,8 | --- | 60,2 |

Standardfehler (geclustert nach Team-ID) in Klammern. * p < 0,10, ** p < 0,05, *** p < 0,01

Quelle: Frick et al. (2020b).

onalen Liga. Für die an der Europa League teilnehmenden Clubs betragen die entsprechenden Werte rund 18 Mio. Euro (Ausgaben) und 12 Mio. Euro (Einnahmen).

Obgleich die vorliegende Schätzung strenggenommen nicht als kausaler Effekt interpretiert werden kann, deuten die Ergebnisse auf einen Zusammenhang der COVID-19-Pandemie und dem veränderten Transverhalten im professionellen Fußball hin. Dies ist nicht zuletzt vor dem Hintergrund der reduzierten Einnahmen der Clubs eine naheliegende Vermutung. Aufgrund der Unsicherheit über die zukünftige Leistungsfähigkeit und Rentabilität sind Spielertransfers durchaus riskante Investitionen (Vöpel, 2006). Kommt zu dieser Unsicherheit die Ungewissheit über die zukünftige Entwicklung der Medien- und Ticketeinnahmen, wird dies zu einer abwartenden Transferpolitik in vielen Vereinen führen. Klubs mit erfolgreichen Trading-Modellen, die auf die Entwicklung und Marktwertsteigerung von (jungen) Spielern setzen, könnten hier besonders betroffen sein. Doch auch niedrigklassige Vereine drohen auf dem Transfermarkt zurückzufallen. Durch die Aussetzung des Ligabetriebs im Amateur- und im semiprofessionellen Bereich werden Spielerverkäufe in höherklassige Ligen erschwert, da die Bewertung der spielerischen Qualität, das sogenannte Scouting, derzeit nicht erfolgen kann. Auch wenn bei Vereinswechseln von Fußballern mit Amateurlizenz keine Ablösesummen anfallen, können diese für Amateurclubs aufgrund von Ausbildungsentschädigungen und Solidaritätszahlungen lukrativ werden.[3] Letztere berechnen sich prozentual zum Ablösewert und können bei kostspieligen Transfers im Laufe der Karriere eines Spielers beachtliche Einnahmen bedeuten, die für niedrigklassige Vereine existenziell sein können. Zudem droht auch hier weiterhin der Ausfall von Spieltagerlösen. Insbesondere in semiprofessionellen Ligen stellt dies eine zusätzliche Bedrohung dar, da häufig ein erheblicher Teil des Umsatzes durch Ticketverkäufe generiert wird.[4] Das finanzielle Ungleichgewicht zwischen Amateur- und professionellen Klubs droht somit weiter zu wachsen. Ähnliche Entwicklungen drohen auch innerhalb der höheren professionellen Ligen, wenn Vereine mit finanzstarken Investoren die durch die COVID-19-Pandemie verursachten Einnahmeausfälle kompensieren bzw. überkompensieren.

3 Neben anfallenden Zahlungen bei der ersten professionellen Vertragsunterschrift (Ausbildungsentschädigungen) fallen auch fortlaufend Solidaritätszahlungen bei Transfers während der Karriere eines Spielers an. Je nach Dauer und durchlaufenen Altersklassen können Ausbildungsvereine bei Spielerwechseln bis zu 5 % der gezahlten Ablösesumme erhalten (FIFA, 2019).

4 Dies gilt insbesondere für zuschauerstarke, semiprofessionelle Ligen wie z. B. die britische National League, die einen größeren Anteil ihres Umsatzes durch Spieltagseinnahmen generieren und deutlich geringere Erlöse durch Marketing und den Verkauf der Übertragungsrechte erzielen als höherklassige Mannschaften (Bridgewater et al., 2020).

## COVID-19-Pandemie erhöht Unsicherheit

Die COVID-19-Pandemie stellt für alle involvierten Akteure zweifellos den größten Stresstest seit dem Beginn der Kommerzialisierung des Fußballsports dar. Die signifikant niedrigeren Transferausgaben und -einnahmen deuten auf ein zurückhaltendes Investitionsverhalten der Vereine hin. Die sorgfältige Analyse weiterer marktspezifischer Indikatoren wird das gesamte finanzielle Ausmaß der Krise deutlich machen. Es bleibt abzuwarten, ob das Ende der Pandemie eine finanzielle Erholung des Marktes und eine Rückkehr zum ursprünglichen Investitionsniveau zur Folge haben wird. Dies wird ferner auch von den derzeit diskutierten Maßnahmen abhängen, den Fußball krisenresistenter und nachhaltiger zu machen (Quitzau und Vöpel, 2020). Diese scheinen unumgänglich, um die zunehmenden ligen- und vereinsspezifischen Ungleichgewichte zu reduzieren und einen ausgeglichenen und spannenden Wettbewerb zu gewährleisten.

Prof. Dr. Bernd Frick ist Inhaber des Lehrstuhls für Organisations-, Medien- und Sportökonomie an der Universität Paderborn.

David Mainus war Projektmitarbeiter am Hamburgischen WeltWirtschaftsInstitut (HWWI).

Paul Schumacher war wissenschaftliche Hilfskraft am Hamburgischen WeltWirtschaftsInstitut (HWWI).


Literatur

Bond, A. J., D. Parnell, P. Widdop und R. Wilson (2020), COVID-19, networks and sport, Managing Sport and Leisure, 1–7.
Bridgewater, S., D. Cockayne und K. Maguire (2020), Written evidence submitted by David Cockayne, Kieran Maguire and Professor Sue Bridgewater–Select Committee Response.
Deutscher Fußball-Bund (2020), 3. Liga–Saisonreport 2019/2020.
Deutsche Fußball Liga (DFL) (Hrsg.) (2009), Stadionhandbuch: Anforderungen an Fußballstadien in baulicher, infrastruktureller, organisatorischer und betrieblicher Hinsicht.
Emrich, E., M. Frenger, F. Follert und L. Richau (2019), Follow me… on the relationship between social media activities and market values in the German Bundesliga, Diskussionspapiere des Europäischen Instituts für Sozioökonomie e.V.
Ernst & Young und Première Ligue (2020), Étude sur l’impact économique et social du COVID-19 sur les clubs de football professionnel de Ligue 1 - Synthèse, premiere-ligue.fr/wp-content/uploads/2020/07/EY-Premiere-Ligue-Impact-COVID-19-Synth%C3%A8se.pdf (25. Januar 2021).
Fédération Internationale de Football Association (FIFA) (Hrsg.) (2019), Reglement bezüglich Status und Transfer von Spielern.
Frick, B., M. Lang, K. Maguire und T. Quansah (2020a), The impact of the coronavirus outbreak (COVID-19) on player salaries, transfer fees, and net transfer expenses in the English Premier League, im Erscheinen.
Frick, B., D. Mainus und P. Schumacher (2020b), Die Auswirkungen von COVID-19 auf die Transferaktivitäten der Fußballvereine der fünf europäischen Top-Ligen im Sommer 2020, HWWI Policy Paper, 130.
Gerhards J Mutz und M Wagner G G Die Berechnung des Siegers: Marktwert, Ungleichheit, Diversität und Routine als Einflussfaktoren auf die Leistung professioneller Fußballteams Zeitschrift für Soziologie 2014 43 3 231 250 10.1515/zfsoz-2014-0305
Panja, T. (2020), Top French Leagues Scrambling After Broadcast Deal Vanishes, New York Times, 15. Dezember.
Quitzau, J. und H. Vöpel (2020), Zwischenruf: Zur Reform des Profifußballs, HWWI Standpunkt, August.
Reuters (Hrsg.) (2020), Mediapro seals agreement over French soccer rights dispute-media, 11. Dezember, reuters.com/article/uk-francesoccer-media-agreement-idUKKBN28L21R (25. Januar 2021).
Union of European Football Associations (UEFA) (Hrsg.) (2020), Country coefficients, uefa.com/memberassociations/uefarankings/country (25. Januar 2021).
Vöpel, H. (2006), Ein Transfermarktmodell und Implikationen für die strategische Transferpolitik der Vereine in der Fußball-Bundesliga, HWWI Research Paper, 1–5.
